# Trigeminal neuralgia: IncobotulinumtoxinA (Xeomin), can it decrease the pharmacological intervention? (A case series)

**DOI:** 10.22088/cjim.14.2.376

**Published:** 2023

**Authors:** Meghdad Hosseini, Farhad Asarzadegan, Erfan Shafiee, Shayan Alijanpour

**Affiliations:** 1 Department of Neurology, Shahid Beheshti University of Medical Sciences, Tehran, Iran; 2 Students Scientific Research Center, School of Nursing and Midwifery, Tehran University of Medical Science, Tehran, Iran; 3 Research and Planning Unite, Pre-hospital Emergency Organization and Emergency Medical Service Center, Babol University of Medical Sciences, Babol, Iran

**Keywords:** Trigeminal, Neuralgia, Pain, Iran.

## Abstract

**Background:**

Trigeminal neuralgia is one of the most disabling facial pain syndromes. In recent years' new therapeutic strategy, incobotulinumtoxin A has emerged. The aim of the current study was to determine the time and duration of pain in 3 cases with pharmacological treatment and incobotulinumtoxin A.

**Case presentation:**

In three patients with different onsets, trigeminal neuralgia was diagnosed. Pain severity was assessed by the visual analogue scale. Patient demographics and clinical data were filled in a checklist. They were females with age ranging from 39 to 49 years. Two patients had normal MRI and one patient had no any recent MRI. One center and specialist injection Xeomin 50 units for one time. Despite long time oral treatment, their symptoms had no significant improvement, but after incobotulinumtoxin A injection, pain frequency, severity and duration decreased in patients***.***

**Conclusion:**

Result showed that the frequency, severity and duration of pain attacks was efficiently decreased by incobotulinumtoxin A with low side-effects. Its complication and side-effect should be considered in the future.

One of the most disabling facial pain syndromes is trigeminal neuralgia (1). The International Association for the Study of Pain(IASP) defines TN as sudden, usually unilateral, severe, brief, stabbing, and recurrent episodes of pain in the distribution of one or more branches of the trigeminal nerve (2). It is affecting the trigeminal nerve and is characterized by severe, paroxysmal, lancinating facial pain, especially in areas where some branches of the nerve are distributed (3). The pain is triggered by a stimulation, such as wind blowing on one’s face, touching the face softly, moving the face and etc. (3). The diagnosis of trigeminal neuralgia is primarily based on history and clinical criteria(4). According to HIS (Headache International Society), it is classified into classical and symptomatic types. The pharmacological intervention by carbamazepine or oxcarbazepine was suggested by the American Academy of Neurology and the European Federation of Neurologic Societies. Although, despite adequate treatment, some patients may experience intractable pain or intolerable side effects that lead to discontinuation (2). Recently, the botulinum toxin administration has shown as a safe strategy in drug-refractory idiopathic trigeminal neuralgia (1). We present the three TN patients treated by incobotulinumtoxin A (Xeomin). It is a formulation of botulinum neurotoxin type A that is free of complexing proteins. Administration was conducted to determine the effect of this intervention on the frequency of facial pain attack.

## Cases presentation

**Figure 1:**
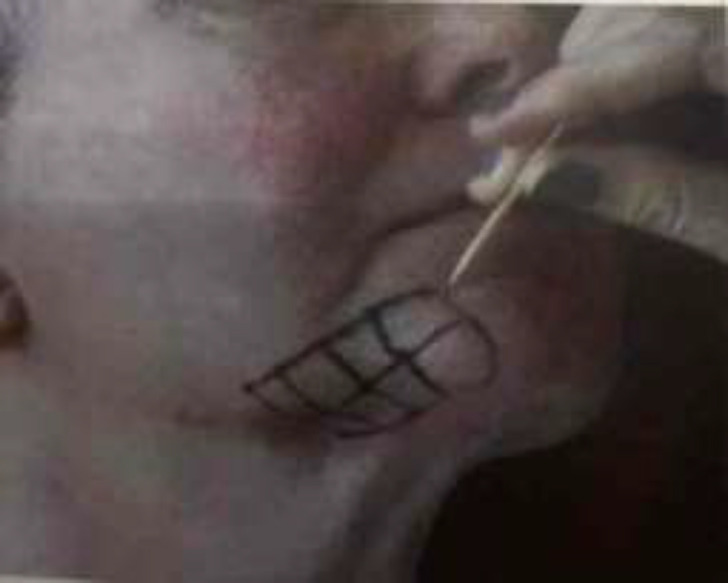
The site of injection in three patients

Three TN patients in 2019 were included in this study and were diagnosed in a private clinic in Tehran, Iran. Rushton and Olafson criteria for the diagnosis of TN was used (3). All these patients were females and age ranged from 39 to 49 years. Two patients had normal MRI and one patient has not had any recent MRI. All procedures were performed in one single center by the same neurologist and patients received Xeomin 50 units for one time as intradermal injection of incobotulinumtoxin A per square centimeter (6). The site of injection was shown in figure 1**. **We used checklist to complete patient's data. It has 11 questions that includes name, age, facial pain period onset, VAS (visual analogue scale) for facial pain(self-report), number and type of drugs used before and after incobotulinumtoxin A administration, complications, number of pain attack before and after the incobotulinumtoxin A and MRI or para-clinical findings.

**Case 1: **A 39-year-old female, a housewife was referred by a neurosurgeon with a history of an 8-year unilateral facial pain and her VAS was 10. Her past history showed she had hypothyroidism for 5 years which is well controlled by levothyroxine. She had a surgery two years ago and had filled the dents. The pain usually started with discomfort and anxiety. First line treatment was stablished with Tegretol 200 mg and Baclofen 10 mg once daily and Phenytoin 100 mg, Fluoxetine 20 mg and Tegretol 400 mg twice daily. Facial pain attack after incobotulinumtoxin A decreased from 4 times a week to once a week. Also, normal findings in MRI were reported. After follow-up (2 years after injection) her Vas was 0.

**Case 2: **A 49-year-old female, a housewife, was referred by her dentist with 5 years of unilateral facial pain, her VAS was 10. Her past history showed that after dental pain and root canal, patient has been suffering from left mandible pain. She had no history of any significant underlying disease. The pain started with discomfort and anxiety, or while eating, talking, or in contact with water and touching. First line treatment began with Tegretol 400 mg twice daily and Amitriptyline 25 mg daily and after incobotulinumtoxin A administration, she received Pregabalin 150 twice daily and Duloxetine 30 mg daily. Facial pain attacks after incobotulinumtoxin A decreased from 20-30 times a week to once a week. Also, normal findings in MRI were reported. After follow-up (2 years after injection) her Vas was 0.

**Case 3: **A 41-year-old female, housewife, was referred with 12 years of unilateral facial pain, and her VAS was 10. Her medical history revealed she had multiple sclerosis for the past 10 years which was diagnosed by MRI and well controlled by treatment. The pain started with fatigue,or while eating, drinking, talking, toothbrushing, in contact with water and touching. First line treatment began with Tegretol 1200 mg daily and Baclofen 10 mg daily and after incobotulinumtoxin A was repeated. After injection, complication was seen as mild paresis on the side of injection. Facial pain attacks after incobotulinumtoxin A administration decreased from 20 times in a week to once a week. Also, after follow-up (2 years after injection) her Vas was 2.

## Discussion

In this study, we expressed three TN cases that received incobotulinumtoxin A. The pain attack and severity of it decreased. Only 1 case had low side-effects. Although, transient whole-body discomfort, mild left eye ptosis, and slight oral deviation were seen in Liu et al.’s study (7).

In Badarny et al.’s study, the mean treatment duration was 10 years, 2441 treatments were administered, injection doses and treatment responses were consistent during the study. No major side effects were reported. Also, relatively few minor adverse events were reported. That was similar with current study. Badarny et al.’s study showed Botulinum toxin (BTX-A) is a satisfactory long-term treatment without need for dose increase over (8). In current study we studied only incobotulinumtoxin A for similarity in the evaluation of outcome of TN patients that was different with Badarny. Low side-effect and effective of administration of incobotulinumtoxin A was similar in two study. In Zhang et al.’s study conducted 84 trigeminal neuralgia patients treated by BoNT type. The response rate was 70% for the 25 unit and 86% for the 75-unit group, both significantly higher than the placebo group (32%). Reduction of pain occurred as early as week 1. Also, higher doses was not necessarily better and could cause more side effects (facial weakness) (9). Furthermore, Apalla et al.’s study assessed the effect of incobotulinumtoxin A (onaA-BoNT). The toxin group met the primary outcome which was 50% or more reduction in VAS score measured at week 4 compared to baseline significantly (10).

A controlled study demonstrated that the therapeutic efficacy of BTX-A was significantly superior. to that of placebo in pain intensity. Open-label trials confirmed this trend. After BTX-A injection, the reduction in the mean pain intensity from baseline was 41-81% at 1 wk, 66-98% at 4 wk, about 80% at 8 wk and 12 wk (11).

A controlled study and open-label trials also demonstrated that the therapeutic efficacy of BTX-A was significantly superior to that of placebo in reducing daily pain frequency. The mean daily attacks were 21-33 at baseline, but 3.6-8.4 at 1 wk, 4.1-4.7 at 4 wk and 1.8-2.3 at 8-12 wk after BTX-A injection. 

Other reported AEs of BTX-A injection included transient edema (2.2%), eyelid ptosis (1.1%), dysesthesia (1.1%) and difficulty in chewing (1.1%). As similar with current study, dysphagia and systemic side effects were not reported in all the 5 trials (12). In Bohluli’s study, patients with complete eradication of the pain were also reported and there was no need for further medication. Although the local or systemic adverse events were not very well reported except transient facial asymmetry (13). In line with other study, a short-term facial weakness was seen in the injection side. The diagnosis of trigeminal neuralgia should be considered in all patients with unilateral facial pain. Some disorders such as cluster headache, dental pain, giant cell arteritis, glossopharyngeal neuralgia, intracranial tumors, migraine, multiple sclerosis, otitis media, paroxysmal hemicrania, postherpetic neuralgia, sinusitis, SUNCT(shorter lasting, unilateral neuralgiform, conjunctival injection, and tearing), temporomandibular joint syndrome and trigeminal neuropathy might be included in the differential diagnosis of trigeminal neuralgia (5).

**In conclusions o**ne of the new therapeutic strategies in TN is incobotulinumtoxin A administration. This study shows that the frequency, severity and duration of facial pain attack efficiently decreased after incobotulinumtoxin A administration. Its complication and side-effect should be considered in the future.
